# Effects of repetition as training and incentives on the performance in pulmonary function tests in healthy volunteers

**DOI:** 10.1016/j.heliyon.2023.e17594

**Published:** 2023-06-22

**Authors:** Julia Krabbe, Annika K. Kotro, Thomas Kraus

**Affiliations:** Institute of Occupational, Social and Environmental Medicine, Medical Faculty, RWTH Aachen University, Pauwelsstraße 30, 52074, Aachen, Germany

**Keywords:** Spirometry, Pulmonary function test, Training, Repeated testing, Coefficient of variance

## Abstract

Pulmonary function testing (PFT) is a central part of diagnosis and treatment monitoring in respiratory medicine. Few studies have investigated whether repeated PFT or training can significantly influence performance.

To investigate potential training effects of repeated PFT, 30 healthy volunteers underwent daily and weekly repeated PFT with spirometry over 10 weeks. The study included 22 females and 8 males with a mean age of 31.8 years ± 15 (SD), a mean weight of 66.3 kg ± 14.5 (SD) and a mean BMI of 22.4 ± 3.3 (SD). The first 5 PFTs were performed on 5 consecutive days, followed by 3 PFTs once a week on the same day of the week. Subsequently, 5 measurements were taken daily for 5 consecutive days. After these 13 appointments in 5 weeks, participants were randomly assigned to the control or incentive group, with stratification for age and gender. The incentive group had the opportunity to win money (200 €) for the highest increase in forced vital capacity (FVC). PFTs were performed once a week on the same day of the week as before for 5 more times. Motivation was assessed by a questionnaire before the 1st, 9th and 18th measure of PFT at three time points throughout the study.

An increase in PFT was observed with mean increases of 473 [ml] in FVC, 395 [ml] in forced expiratory volume in 1 s (FEV1) and 1.382 [litres/second] in peak expiratory flow (PEF) after four days of daily PFT. These increases did not persist and spirometric data returned to baseline after one week. After allocation, participants in the incentive group did not increase their FVC, FEV1 or PEF compared to the control group. The incentive group showed higher motivation than the control group, even before allocation.

Repeated daily PFT could induce short-term increases, but PFT does not fluctuate significantly in the long term. External influences that affect motivation could not consistently increase PFT. For clinical practice, it can be concluded that PFT does not necessarily require extended training to ensure reliability if reproducibility criteria are met.

## 1 Introduction

Pulmonary function testing (PFT) is an essential part of the diagnosis and monitoring of lung disease. As a non-invasive technique, it is frequently used, sometimes several times at short intervals, e.g. as part of close monitoring of therapy.

According to the European Respiratory Society/American Thoracic Society (ERS/ATS) guidelines [1], a deviation of less than 5% for forced expiratory volume in 1 s (FEV1) and forced vital capacity (FVC) between the best and second best of at least three maneuvers is required to meet the reproducibility criteria. Patients are instructed to exert maximum effort during maneuvers to ensure reproducibility between maneuvers and between different appointments. However, familiarity with the PFT procedure and maneuver may influence the ability to recall maximal effort.

In general, the learning effects of PFT repetition appear to be small [2,3], while some studies have found no effects at all [4]. Larsson and colleagues found rather small effects depending on the time of day, but no learning effects [5]. Circadian effects might be responsible for the small differences with the highest scores between noon and afternoon [6].

To investigate intraindividual differences between subjects, the coefficient of variation (CoV) for lung function parameters can be compared as the ratio of the standard deviation to the mean. Coefficients between 2 and 8% are regularly reported [7,8], but some studies have observed an inverse correlation between the coefficient and adherence in chronic obstructive pulmonary disease (COPD) [9], asthma control [10] or clinical outcome in cystic fibrosis [11]. Here, CoV is not used as a measure of reproducibility.

In general, there is a paucity of literature on the variability of repeated PFTs in healthy subjects at short intervals. In occupational medicine, PFT is regularly used in the workplace, e.g. for the detection of work-related asthma, and longitudinal studies are crucial for the early detection of respiratory diseases. It is therefore important to have high quality PFT methods that are as sensitive as possible.

Based on the current state of the literature and personal experience with increasing FVC by repeated daily PFT, it was hypothesized that repeating PFT over several days would result in a small increase in FVC. It was assumed that no further voluntary increase would be detectable after possible training effects. 30 healthy volunteers performed daily and weekly repetition tests. In addition, the effects of an incentive were investigated and motivation was determined using a questionnaire.

The aim of this study was to discover relatively large effects that would have a relevant influence on the results and interpretation of repeated PFTs after short intervals in daily clinical practice.

## 2 Material and methods

### 2.1 Study design

The study was carried out between January and May 2022. Participants were recruited through postings on local and online bulletin boards, mainly in university buildings, the university hospital and the university sports center. They were paid €50 to participate and were informed that an additional €200 would be raffled among participants at the end of the study.

**Fig. 1:**
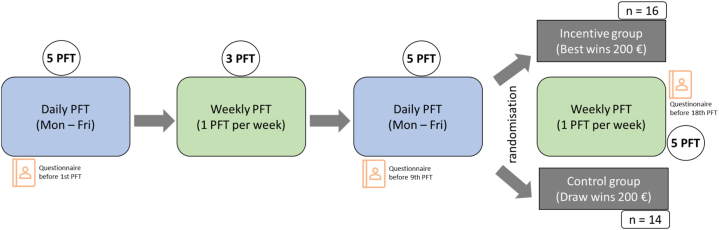
**Scheme of study protocol**: After daily PFT in the first week, three weeks with weekly PFT always on the same day of the week and another week with daily PFT followed. Afterwards, participants were then randomly assigned to the incentive group or the control group. Both continued weekly PFT for another five weeks. In the control group 200 € were raffled among the group. In the incentive group 200 € were awarded to the person with the highest increase in FVC. All participants received 50 € for participation. A motivation questionnaire was filled out before measures 1, 9 and 18.

The study protocol was as follows (Fig. 1).

The first week consisted of five PFT with spirometry from Monday to Friday. To eliminate circadian effects, the PFT was performed at the same time (around noon or afternoon) for each participant. After the first week, testing continued once a week for the next three weeks, always on the same day of the week. This was followed by another week of daily testing. After these 13 appointments, participants were randomly assigned to one of two groups, with stratification by gender and age: 1) control group and 2) incentive group. In the control group, weekly testing continued for 5 weeks, and at the end, €200 was raffled among the group for one participant. In the incentive group, participants were informed after the PFT on Friday (13th test) that they were now part of a competition. The person who improved their FVC the most would win €200. After each appointment they were informed whether they had increased their FVC or not. The predicted FVC/FVC percentage (FVC%) was used to determine the personal increase and to decide who would win the competition. To ensure that all potential participants were aware of the opportunity to win €200, information about the chance to win 200€ was given prior to informed consent and enrolment.

If a participant missed an appointment, the PFT was performed on the following day or one week later on the same day. Missing more than two appointments would have resulted in exclusion from the study, but this did not occur.

To account for effects on motivation, a questionnaire was given at three appointments: 1, 9 and the last. The questionnaire was adapted from the Questionnaire on Current Motivation (QCM) by Rheinberg and colleagues [12] in German. It consisted of 8 questions with a 7-point rating scale from strongly disagree to agree. A translated version (adapted from Ref. [13]) of the questionnaire is provided in supplementary file 1. Items were selected to assess the motivational factors of challenge (items 1, 5 and 7), interest (items 3 and 8), anxiety (items 2 and 6) and likelihood of success (item 4).

An overall score was calculated for the six items with a positive response pattern (items 1, 3–5, 7, 8) and was formed for each time point.

#### 2.1.1 Pulmonary function test (PFT)

All PFTs were performed by the same instructor using a commercial device (Vyaire medical, MasterScreen, Hoechberg, Germany) at the Institute of Occupational, Social and Environmental Medicine, RWTH Aachen University Hospital. Spirometric data were derived from a maneuver sequence including slow deep expiration, maximal inhalation and forced expiration, following the ERS guidelines for standardization of spirometry [14] and technical standards for interpretation strategies [1]. The reference values provided by the Global Lung Function Initiative (GLI) 2012 reference equations [15] were used to normalize the obtained lung function data.

#### 2.1.2 Participants

37 healthy volunteers were initially enrolled in this study. Inclusion criteria were age 18 years or older and being a non-smoker, including never smokers or ex-smokers for at least 12 months with a maximum cumulative smoking status of 10 pack-years. Exclusion criteria were any history of asthma or other pulmonary or cardiac disease, previous PFT with evidence of obstructive or restrictive ventilation patterns, as well as incomplete coronavirus disease 2019 (COVID19) vaccination status at that time, previous severe acute respiratory syndrome coronavirus type 2 (Sars-CoV-2) infection, or pregnancy/breastfeeding.

All 37 participants were screened for inclusion and exclusion criteria on the first study day prior to PFT. Height and weight, as well as previous history of PFT and frequency of exercise (endurance and strength training per week) were recorded. Typical reasons for PFT prior to this study included regular childhood PFTs with no current medical condition, annual testing for medical examinations for diving sports, or even one PFT prior for medical examinations due to employment or other study involvement.

The first PFT was used to screen for abnormalities and values below the lower limit of normal (LLN). If none were found, it served as the first PFT for the study. No other diagnostic tests, such as spiroergometry, were used.

Two participants were excluded from the study during the test periods due to the development of an obstructive ventilation pattern or COVID-19 with pulmonary symptoms, in this case cough. One participant dropped out without further explanation. 34 subjects completed the study. All had spirometry data above the lower limit of normal. Before statistical analysis, an outlier analysis was performed, and four participants were excluded. Finally, 30 participants were included in the statistical analysis.

The study was approved by the Ethics Committee of the Medical Faculty of RWTH Aachen University (EK 364-21). All subjects gave written informed consent prior to enrolment.

The characteristics of the participants and the baseline data of the PFT are shown in Table 1, Table 2. The detailed characteristics of each participant can be found in supplementary file 2.

**Table 1:** Anthropometric data of study participants overall, in control and incentive group.

		Groups	
	Overall	control	incentive
	n (%)		
men	8 (26.7)	5 (30.8)	3 (18.7)
women	22 (73.3)	9 (69.2)	13 (81.3)
total	30	14	16
	median (range)		
age (years)	24 (20–66)	23 (20–59)	27 (21–66)
height (m)	1.70 (1.52–1.80)	1.70 (1.52–2.00)	1.70 (1.58–1.87)
weight (kg)	60 (49–108)	61 (49–94)	59 (54–108)
Body Mass Index (BMI)	21.2 (17.7–30.9)	20.7 (17.7–28.3)	22 (18.7–30.9)
endurance training (times per week)	2 (0–6)	2 (0–6)	2 (0–4)
strength training (times per week)	0 (0–4)	0 (0–4)	0 (0–4)
	n (%)		
PFT before	18 (60)	8 (57.1)	10 (62.5)

**Table 2:** Baseline values of spirometry of study participants overall, in control and incentive group.

Pulmonary function testing - Baseline				
	**Overall**	control	incentive	
	mean (±SD)			p value
FVC (liter)	4.56 (1.06)	4.59 (1.05)	4.53 (1.09)	0.8903
FVC (%pred)	105.17 (11.68)	102.14 (8.07)	107.82 (13.70)	0.1895
FEV1 (liter)	3.75 (0.94)	3.79 (0.89)	3.72 (0.95)	0.8397
FEV1 (%pred)	102.77 (14.44)	98.93 (9.71)	106.13 (17.10)	0.1776
PEF (liter/sec)	7.64 (2.16)	7.58 (2.32)	7.69 (1.94)	0.8880
PEF (%pred)	97.09 (18.75)	91.74 (15.53)	101.77 (20.88)	0.1516

Nutritional status was not monitored during the study. On the days the questionnaire was administered (9th and 18th PFT measurement) participants were asked if there had been any changes in weight or exercise since the last questionnaire. Changes were recorded. During the 18 weeks of the study, there were no significant changes in weight or exercise. Weight change occurred for a few participants but only ± 1–2 kg.

### 2.2 Statistical analysis

For sample size calculation, for differences in FVC with an alpha error of 0.05 and power of at least 80%, 45 cases were needed to detect medium effects (d = 0.5) and 19 cases to detect large effects (d = 0.8). The analysis was performed using G Power version 3.1.9.6 [16,17].

Thus, we aimed to recruit approximately 50 participants. However, due to the amount of time required and the ongoing pandemic situation with the omicron variant of the Sars-Cov2 virus, we did not achieve this number of participants and were satisfied to be able to detect at least large effects due to training.

**Fig. 2:**
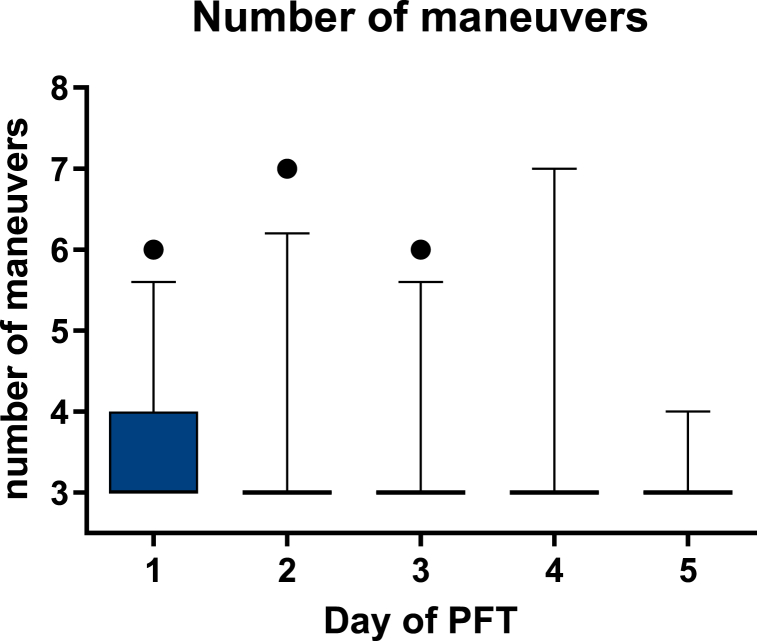
**Number of maneuvers necessary to fulfil reproducibility criterium:** Deviation of test values for FEV1 and FVC <5% between best and second-best measured value for at least three maneuvers according to ERS/ATS guidelines [1]. Daily PFT first week, n = 30.

**Fig. 3:**
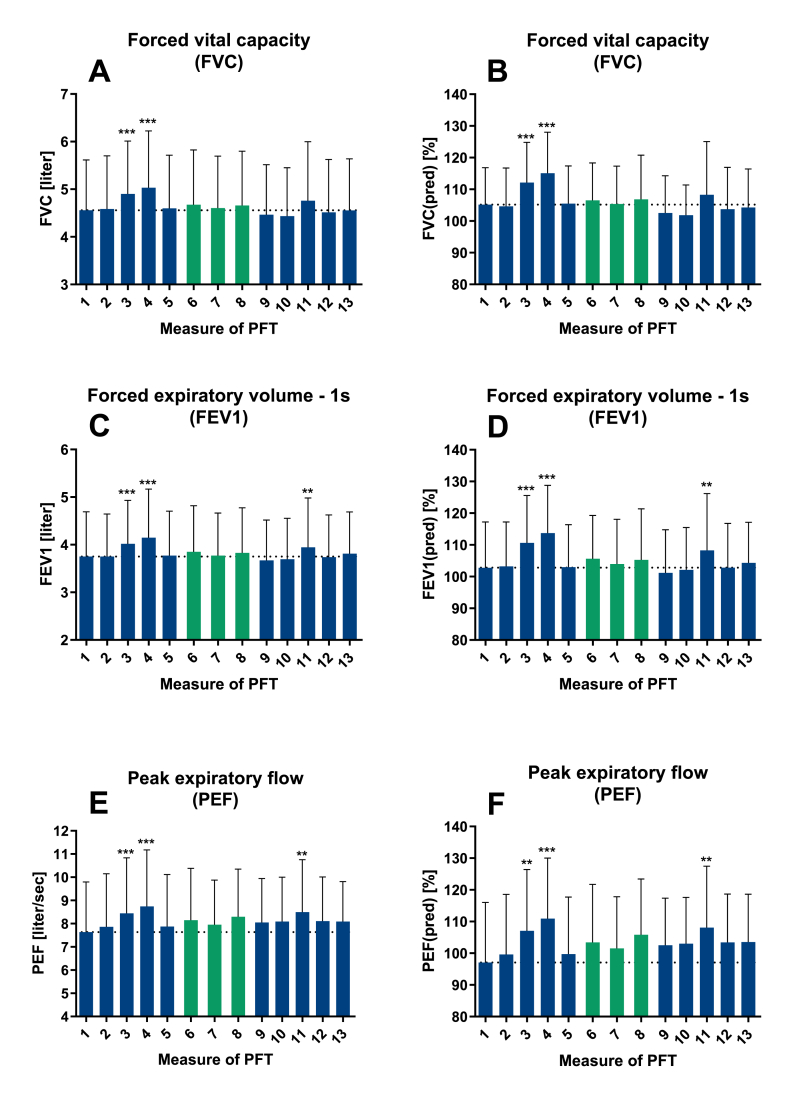
**Effect of training on PFT**: PFT daily (light grey) and weekly (dark grey) over 5 weeks for FVC (A), FVC% (FVC/FVC predicted) (B), FEV1 (C), FEV1% (FEV1/FEV1 predicted) (D), PEF (E) and PEF% (PEF/PEF predicted) (F). The dotted line indicates the mean of measure 1 of PFT. Mean ± SEM, n = 30, ** = p < 0.01, *** = p < 0.001 for comparison with day 1.

**Fig. 4:**
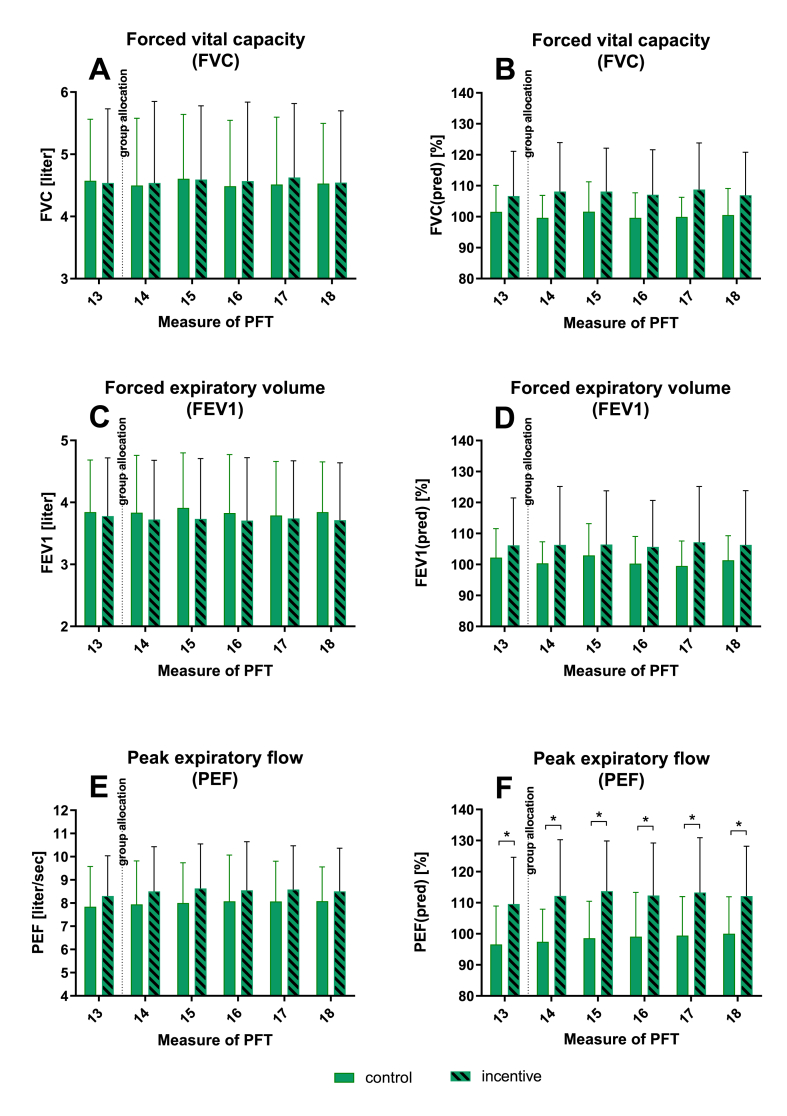
**Differences between control and incentive groups in PFT**: PFT weekly over 6 weeks for FVC (A), FVC% (FVC/FVC predicted) (B), FEV1 (C), FEV1% (FEV1/FEV1 predicted) (D), PEF (E) and PEF% (PEF/PEF predicted) (F). Mean ± SEM, n = 30, * = p < 0.05, ** = p < 0.01. The dotted line indicates time of group allocation.

**Fig. 5:**
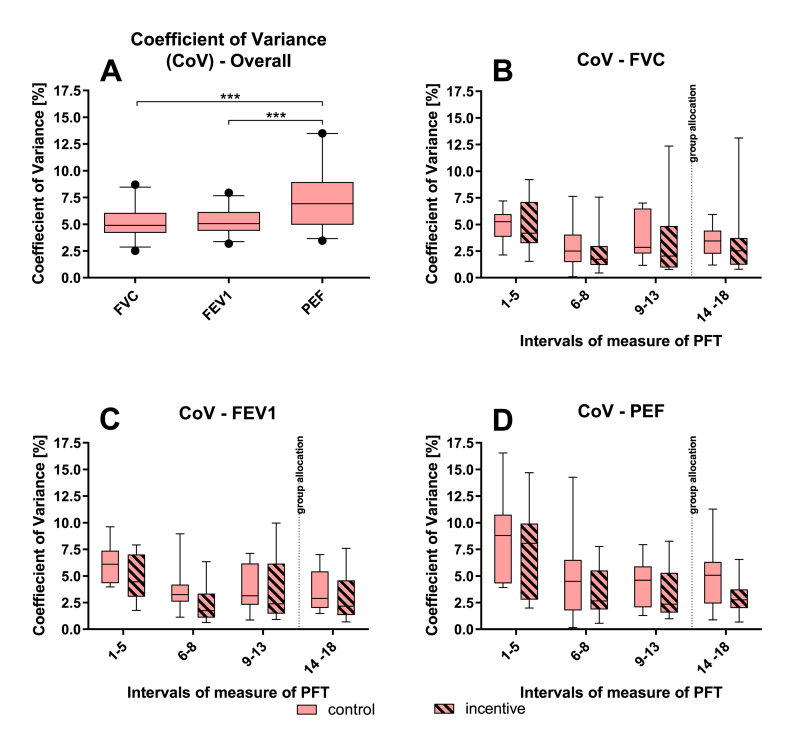
**Coefficient of Variance (CoV)**: CoV over the entire testing period for FVC, FEV1 and PEF (A). CoV for 4 intervals with daily (1–5 and 9–13) and weekly (6–8 and 14–18) PFT for FVC (B), FEV1 (C) and PEF (D). Median with 5th - 95th percentiles, n = 30, * = p < 0.05. The dotted line indicates time of group allocation.

**Fig. 6:**
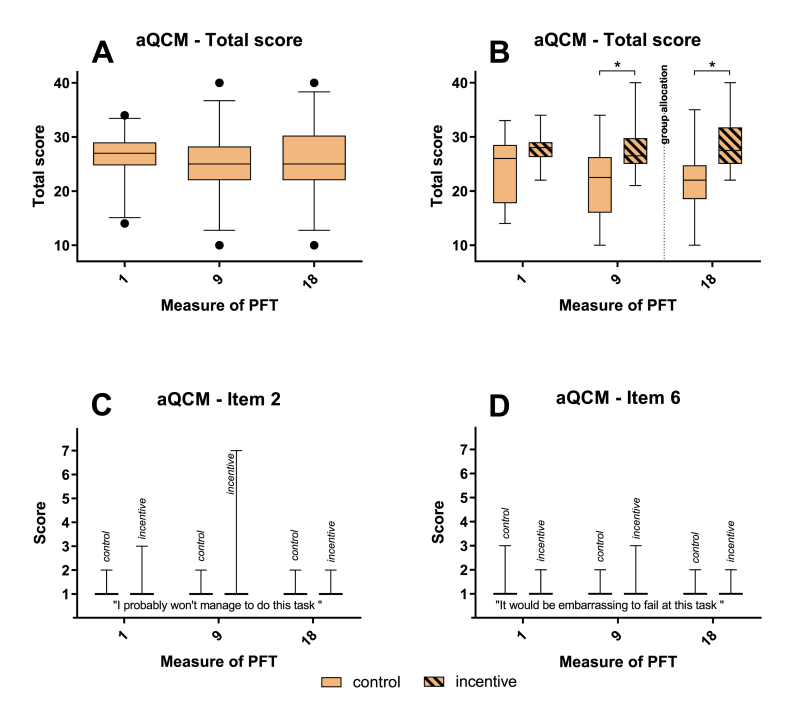
**Effect of training and incentive on motivation**: aQCM questionnaire at first, 9th and 18th measure of PFTs. Total score over 6 positive items for all participants (A) and for groups (B). Item 2 (C) and item 6 (D) were not part of the total score. Median with 5th - 95th percentile, n = 30, * = p < 0.05. The dotted line indicates time of group allocation.

Data analysis was performed using GraphPad Prism 6 (GraphPad, La Jolla, CA). For outlier analysis, the ROUT method (robust regression and outlier removal) [18] with coefficient Q = 0.1% (strict threshold) was combined with kinetic analysis. All data are presented as number of participants (n) and their relative amount (%), median with range and mean with standard deviation (SD) (Table 1, Table 2, Fig. 3, Fig. 4), and box plots with median with 5th - 95th percentiles (Fig. 2, Fig. 5, Fig. 6). Data analysis for comparisons of three or more groups was performed using the Friedman test with post hoc Dunn's test (Fig. 3). Comparisons between two groups were made using unpaired t-tests (Fig. 4, Fig. 5, Fig. 6). Differences in analyses were considered significant at p < 0.05 for two-tailed tests.

## 3 Results

### 3.1 Effects of training on PFT

In general, the number of maneuvers necessary to fulfil the ERS/ATS guidelines reproducibility criteria, a deviation <5% between the best and second-best value of FEV1 and FVC of at least the maneuvers, was low with a mean of 3.4 ± 0.71 (SD) number of maneuvers at day 1 of PFT and a mean of 3.03 ± 0.18 (SD) at the last day.

For day 1 of PFT the 25th - 75^Th^ percentile box of boxplots includes the range from 3 to 4 number of maneuvers, while for the following days no box could be depicted due to small range (Fig. 2).

In the first week of daily PFT an increase with maximum increase in FVC, FEV1 and peak expiratory flow (PEF) on measure 4 of PFT was observed (Fig. 3). This increase did not persist over weekly PFT afterwards. For measure 6–8 of PFT, an increase only for the third measure of daily PFTs could be observed afterwards. However, this increase did not last either (Fig. 3).

For FVC significant differences from measure 1 occurred on measure 3 and 4 of PFT (Fig. 3A). For these measures a difference between means of 343 ml for measure 3 and of 473 ml for measure 4 were registered. For measure 3 and 4 compared to measure 1 of PFT differences of 6.9% and 9.9% respectively could be observed for FVC% (Fig. 3B). For FEV1 and FEV1/FEV1 predicted in percent (FEV1%) significant differences compared to measure 1 of PFT could be observed for measure 3, 4 and 11 (Fig. 3C + D). For these measures differences between means of 295 ml for day 3, 395 ml for measure 4 and 193 ml for measure 11 were registered for FEV1 and 7.8%, 10.9% and 5,5% respectively for FEV1%. Similarly, significant differences compared to measure 1 of PFT could be observed for PEF and PEF/PEF predicted in percent (PEF%) on measure 3, 4 and 11 (Fig. 3E + F). Here, differences between means of 0.81 L/s for day 3, 1.382 L/s for measure 4 and 0.864 L/s for measure 11 were registered for PEF and 10.0%, 13.8% and 11.0% for PEF% respectively.

### 3.2 Effects of an incentive on PFT

After allocation to control and incentive group no significant differences between both groups could be observed for FVC, FEV1, PEF, FVC% and FEV1% for 5 weekly PFT (Fig. 4A–E). For PEF% measure 13 (before allocation) and all measures of PFTs after group allocation showed differences between groups (Fig. 4F) with the highest difference of means of 15.2% for measure 15 of PFT.

### 3.3 Coefficient of variance (CoV)

The CoV over all 18 PFT (10 weeks) was 5.18% ± 1.49 (SD) for FVC, 5.24% ± 1.19 (SD) for FEV1 and 7.42% ± 2,74 (SD) for PEF with PEF significantly differing from FVC and FEV1 (Fig. 5A). To assess the CoV over the course of testing for both groups 4 intervals with daily (measures 1–5 and 9–13) and weekly (measures 6–8 and 14–18) PFT were formed for both groups before and after group allocation and compared. No significant differences could be observed for FVC (Fig. 5B), FEV1 (Fig. 5C) and PEF (Fig. 5D).

### 3.4 Motivation – adapted questionnaire on current motivation (aQCM)

For the total score as sum of all items with positive response pattern (6 items) no significant differences could be observed between time points (Fig. 6A) indicating a constant level of motivation. Generally, motivation was rather high with a mean score of 26.07 ± 4.79 (SD) for day 1, 24.97 ± 6.16 (SD) for day 9 and 25.63 ± 6.63 (SD) for the last day with a possible minimal score of 6 and a maximal score of 42. For the comparison of groups, the total score was higher for the incentive group than the control group for the last two time points before and after group allocation (Fig. 6B). For the items not included in total score (items 2 and 6) no significant differences between groups could be observed (Fig. 6C + D).

## 4 Discussion

Although instruction in PFT should aim for maximum effort from the person undergoing PFT, learning effects of repetition and training effects have been reported. In this study, a remarkable increase in measured volumes was observed with a mean increase in FVC of almost half a liter after four days of daily PFT. Interestingly, these increases were not sustained and spirometric data returned to baseline after one week. After allocation to the control and incentive groups, participants who could win prize money for the highest increase failed to increase FVC, FEV1 or PEF. They showed higher motivation than controls before and after group allocation. CoV was higher for PEF than for FVC and FEV1 but decreased over the course of the study.

This study was inspired by a series of diffusion capacity tests (DCT) over 4 days as part of pre-testing for another study. There we observed a significant increase in alveolar volume (VA) on 4 consecutive days of DCT, with some impact on the interpretation of diffusion capacity (DLCO) and transfer coefficient (Kco) as DLCO/VA. As a review of the literature did not clarify whether this was a known phenomenon and whether this short-term training would persist with repetition, this study was designed as a proof of concept. The first part consisted of 5 sessions of daily PFT training followed by a weekly PFT as a maintenance interval and to assess the persistence of any effects. This was followed by another intensive daily PFT session to test for additive training effects. Consistent with our observations on DCT, FVC, FEV1 and PEF were significantly increased on the first four days with a decrease on the last day of the week. This was most likely due to increasing familiarity with the PFT procedure and the specific maneuver. Accordingly, the number of maneuvers required to meet the reproducibility criterion of deviation of test values for FEV1 and FVC <5% between the best and second best measured value for at least three maneuvers according to the ERS/ATS guidelines [1] was between 3 and 4 on the first day and close to 3 on the fifth day, indicating the increasing ability of the participants to perform. However, the increase of almost 500 mL in FVC and almost 10% in FVC% was not constant, ruling out a permanent learning effect over weeks. If familiarity with the maneuver was the only factor for this observation, a constant effect over several weeks would have been expected, as the interval of one week between PFTs is not long enough to allow for memory fading. Furthermore, motivation did not differ between the first day of PFT and the first day of the second week of daily PFT (day 9), but only a slight increase was observed in week 5 of PFT. However, effects of acclimatization to PFT, instructor and location cannot be excluded as factors influencing motivation that were not captured by the aQCM.

Accordingly, other studies have found significant learning effects for short intervals of minutes to hours between PFTs [3], but not for weeks [5] and months [4,19]. Interestingly, two studies found changes with time of day [5,6] and one did not [20]. These small changes, with increases around midday [6] and decreases towards the afternoon [5,6], have been attributed to circadian rhythm. However, many same-day PFTs could also be considered as training within a day, resulting in increases similar to those observed in the current study. This possible training effect resulted in increased TLC (but unchanged FVC) in one study [5] and persistently increased PEF in another [6], confirming short-term training effects of repeated PFT. Accordingly, a study with PFT intervals comparable to the current study of five PFTs on five consecutive days reported similar increases in FVC and FEV1 in 15 healthy volunteers [21].

The CoVs for FVC and FEV1 found in this study were comparable to CoVs reported in other studies [2,7,8,21]. Intervals between included PFTs ranged from seconds as the interval between consecutive tests [7] to days [21] to months [8]. Interestingly, in the current study, CoV was higher for PEF than for FVC and FEV1. As only healthy subjects with PFT data above the LLN were included, no apparent diseases could have influenced PEF during the study. Furthermore, CoV for FVC, FEV1 and PEF decreased over time, indicating increasing familiarity with the maneuver and ability to perform PFT reproducibly. As an indicator of effort [14], a higher CoV of PEF in the first week of the study indicates that participants did not initially perform at maximal effort. Compared to other studies with PFT intervals of minutes [7], hours [20] and weeks [8], the CoV of PEF in the current study was significantly higher. However, it decreased over the course of the study to comparable values with increasing reproducibility over time.

As participants were able to increase FVC, FEV1 and PEF twice within a four-week interval, it might be expected that the incentive group, who were competing for the highest increase in FVC, would increase significantly again. However, no additional increases were observed after group allocation. This is somewhat surprising. The inability to voluntarily increase their spirometric volumes could indicate that the training had been completed in terms of familiarity with the maneuver. Indeed, after two periods of daily PFT for one week, with three weeks in between, one would expect the participants to have exhausted their training. In the motivation questionnaire, participants in the incentive group already showed higher motivation than controls before and after allocation, suggesting no direct motivational effect of the incentive. The constant feedback of whether they had increased their FVC compared to day 13 should have given participants the opportunity to improve their performance significantly.

Best to our knowledge, most studies assessing training or feedback effects have focused on the instructor [22,23]. In this study, the same instructor performed all PFTs, minimizing instructor-related effects. Nevertheless, the persistence of FVC, FEV1 and PEF over the course of the 10-week study demonstrates the robustness of PFT over time. Despite multiple training and incentive interventions, measures fluctuated around baseline. Future studies should further investigate whether other factors could have a greater impact without violating the reproducibility rules, e.g. efforts to obtain compensation with the desire to underperform.

Age-related factors may have influenced the effects observed in this study. Therefore, stratified randomized allocation was used for group allocation to minimize differences in age and gender between groups. Most participants in both groups were between 20 and 30 years old, and both included participants over 50 years old. There were slightly more older participants in the incentive group, but there was also a slightly higher proportion of participants who had done PFT before. These imbalances were due to dropouts and the stratified but still random allocation. There were no extreme differences between the groups, which at least suggests that these differences did not have a significant effect. However, as age-related decreases in PFT have been described [24], age-related effects cannot be excluded. A higher number of older participants may have masked possible increases in younger participants. As the number of older participants was rather small, this effect would have been negligible for general training effects. However, there were more participants older than 30 years in the incentive group and this could be an additional explanation, apart from the small sample size, for the finding of no difference between groups after allocation.

As noted above, the sample size analysis indicated a larger target sample size to detect medium effects, but with the ongoing pandemic and a difficult recruitment process due to the required 10-week commitment, only the reported 37 participants could be recruited initially. Therefore, due to the small sample size of this study, conclusions must be drawn with caution and are not fully generalizable. Especially for the differences in motivation, the sample size was too small to detect medium differences. Many participants in the incentive group were excited about the possibility of winning the money during the study, but we could not detect large differences between the control and incentive groups.

Regarding weight and health, using BMI alone, three participants in the control group and four in the incentive group were considered overweight (BMI >25), and one participant in the incentive group was considered obese (BMI >30). However, most of our participants were quite athletic and exercised regularly. Thus, most participants with a BMI above 25 and 30, who were formally overweight and obese, reported frequent physical activity and did not seem to be subjectively restricted by their weight or subjectively overweight. This may be due, at least in part, to the general problem of BMI, as it does not take into account whether weight is made up of fat or muscle, and a lower BMI due to lower muscle mass does not indicate health or well-being [25]. As most participants with a BMI over 25 reported regular physical activity, the number of participants with lower muscle mass should have been small. However, this is a limitation as body fat and muscle mass were not measured and cannot fully account for possible effects.

Future studies should investigate the observed effects and consider the limitations of this study: a significantly larger number of participants, ideally with subgroups arranged or divided according to age, sex and BMI or amount of body fat and muscle mass. A more detailed characterization of the participants, e.g. determination of maximal oxygen consumption (VO2max), nutritional status or metabolic equivalent of tasks (METs) could be included if feasible.

This study is one of the few to examine repeated PFTs over days and weeks in healthy subjects rather than patients with respiratory disease. It provides insight into the effects of short-term repetition of PFT beyond medical diagnoses and demonstrates the robustness of PFT and its results to external confounders. For clinical practice, it can be concluded that patients performing PFT for the first time may benefit from repetition of maneuvers, but do not need extended training.

In conclusion, repeated daily PFT can induce short-term increases, but in PFT FVC, FEV1 and PEF do not fluctuate significantly in the long term. Even external influences that affect motivation cannot consistently change PFT. For clinical practice, it can be concluded that PFT does not necessarily require extensive training to ensure reliability, as long as reproducibility criteria are met. In our study with healthy volunteers, personal motivation seemed to play only a minor role in PFT performance. If motivational effects are found in a larger study group, this could be addressed in future studies.

## Author contributions

Conceptualization, JK, AK, TK; Data curation, JK, AK; Formal analysis, JK, AK; Investigation, JK, AK; Methodology, JK, TK; Resources, TK; Supervision, JK; Visualization, JK, AK; Data Curation JK, AK; Writing – original draft, JK, AK; Writing – review & editing, JK, AK, TK.

## Funding

This research did not receive any specific grant from funding agencies in the public, commercial, or not-for-profit sectors.

## Institutional review board statement

All subjects gave their informed consent for inclusion in the study. The study was conducted in accordance with the Declaration of Helsinki and the protocols were approved by the Ethics Committee of the Medical Faculty, RWTH Aachen University (EK 364-21).

## Informed consent statement

Informed consent was obtained from all subjects involved in the study.

## Data availability statement

The datasets generated during and/or analyzed during the current study are available from the corresponding author on reasonable request.

## Declaration of competing interest

The authors declare that they have no known competing financial interests or personal relationships that could have appeared to influence the work reported in this paper.
