# Supplementary material for: Effects of repetition as training and incentives on the performance in pulmonary function tests in healthy volunteers
Source: Heliyon. 2023 Jun 22;9(6):e17594. doi: 10.1016/j.heliyon.2023.e17594 (PMC10319240; doi:10.1016/j.heliyon.2023.e17594)
Supplement: Multimedia component 1 [file mmc1.pdf]

Effects of repetition as training and incentives on the performance in pulmonary function tests in healthy volunteers

Julia Krabbe[1]; Annika K. Kotro[1]; Thomas Kraus[1]

[1]Institute of Occupational, Social and Environmental Medicine, Medical Faculty, RWTH Aachen University, Pauwelsstraße 30, 52074 Aachen, Germany

Suppl. File 1: Motivation questionnaire – English translation. The questionnaire was handed out in German.

Motivation questionnaire

This questionnaire captures your current attitude towards the described task. You will find statements on this page. Please mark the number that fits best.

Item		Disagree						Agree
**1**	I am eager to see how I will perform in the task	1	2	3	4	5	6	7
**2**	I probably won't manage to do this task	1	2	3	4	5	6	7
**3**	In the task, I like the role of scientists who discover connections	1	2	3	4	5	6	7
**4**	I feel under pressure to do this task well	1	2	3	4	5	6	7
**5**	This task is a real challenge for me.	1	2	3	4	5	6	7
**6**	It would be embarrassing to fail at this task	1	2	3	4	5	6	7
**7**	I am really going to try as hard as I can on this task	1	2	3	4	5	6	7
**8**	For tasks like this I do not need a reward, they are lots of fun anyhow	1	2	3	4	5	6	7
