# Supplementary material for: Effects of repetition as training and incentives on the performance in pulmonary function tests in healthy volunteers
Source: Heliyon. 2023 Jun 22;9(6):e17594. doi: 10.1016/j.heliyon.2023.e17594 (PMC10319240; doi:10.1016/j.heliyon.2023.e17594)
Supplement: Multimedia component 2 [file mmc2.pdf]

# Effects of repetition as training and incentives on the performance in pulmonary function tests in healthy volunteers

Julia Krabbe[1]; Annika K. Kotro[1]; Thomas Kraus[1]

[1]Institute of Occupational, Social and Environmental Medicine, Medical Faculty, RWTH Aachen University, Pauwelsstraße 30, 52074 Aachen, Germany

**Suppl. File 2: Detailed characteristics of both study groups**

| Groups | | | | | | | | | | | | | | | |
|---|---|---|---|---|---|---|---|---|---|---|---|---|---|---|---|
| control | | | | | | | | incentive | | | | | | | |
| age (years) | height (m) | weight (kg) | BMI | gender | PFT before | strength training (times per week) | endurance training (times per week) | age (years) | height (m) | weight (kg) | BMI | gender | PFT before | strength training (times per week) | endurance training (times per week) |
| 20 | 178 | 65 | 20.52 | m | no | 0 | 2 | 21 | 185 | 88 | 25.71 | m | no | 4 | 0 |
| 20 | 181 | 58 | 17.70 | m | yes | 0 | 0 | 22 | 168 | 56 | 19.84 | w | yes | 2 | 3 |
| 22 | 160 | 55 | 21.48 | w | yes | 0 | 4 | 22 | 170 | 58 | 20.07 | w | yes | 0 | 2 |
| 22 | 170 | 58 | 20.07 | w | yes | 1 | 0 | 23 | 164 | 58 | 21.56 | w | yes | 3 | 3 |
| 23 | 160 | 49 | 19.14 | w | no | 0 | 1 | 23 | 178 | 79 | 24.93 | w | yes | 0 | 2 |
| 23 | 163 | 52 | 19.57 | w | yes | 2 | 0 | 24 | 158 | 56 | 22.43 | w | no | 0 | 3 |
| 23 | 173 | 79 | 26.40 | m | no | 0 | 1 | 24 | 173 | 75 | 25.06 | w | no | 0 | 3 |
| 23 | 176 | 63 | 20.34 | w | no | 0 | 3 | 27 | 170 | 56 | 19.38 | w | yes | 0 | 1 |
| 24 | 152 | 50 | 21.64 | w | yes | 2 | 2 | 27 | 187 | 108 | 30.88 | m | no | 1 | 3 |
| 24 | 176 | 65 | 20.98 | m | no | 4 | 2 | 31 | 170 | 54 | 18.69 | w | no | 0 | 2 |
| 25 | 200 | 94 | 23.50 | m | no | 0 | 6 | 36 | 165 | 73 | 26.81 | w | yes | 0 | 1 |
| 27 | 170 | 59 | 20.42 | w | yes | 0 | 6 | 40 | 168 | 65 | 23.03 | w | yes | 1 | 4 |
| 50 | 168 | 80 | 28.34 | w | yes | 0 | 3 | 55 | 164 | 55 | 20.45 | w | no | 0 | 4 |
| 59 | 167 | 77 | 27.61 | w | yes | 0 | 0 | 64 | 173 | 60 | 20.05 | w | yes | 0 | 0 |
| | | | | | | | | 64 | 186 | 87 | 25.15 | m | yes | 0 | 0 |
| | | | | | | | | 66 | 168 | 56 | 19.84 | w | yes | 2 | 1 |
